# A Formal Representation for Numerical Data Presented in Published Clinical Trial Reports

**Published:** 2013

**Authors:** Maurine Tong, William Hsu, Ricky K Taira

**Affiliations:** a Department of Bioengineering, University of California, Los Angeles; b Medical Imaging Informatics Group, Department of Radiological Sciences, University of California, Los Angeles

**Keywords:** Knowledge representation, visualization, data analysis, data interpretation, clinical trial

## Abstract

Assessing the quality of and integrating clinical trial reports are necessary to practice evidence-based medicine. In particular, the numerical data is essential to understanding the strength and quality of the clinical trial study. In this paper, we present a formal representation for standardizing numerical data in published clinical trial reports, and our efforts towards developing computational tools to capture and visualize this representation. The approach includes two aspects: a process model used to precisely define experimental context behind the numerical value; and a spreadsheet, an intuitive and familiar tool used to organize numerical data. We demonstrated this representation using clinical trial reports on non-small cell lung cancer (NSCLC). We performed a preliminary evaluation to determine the usefulness of this formalism for identifying the characteristics, quality and significance of a clinical trial. Our initial results demonstrate that the representation is sufficiently expressive to capture reported numerical information in published papers.

## Introduction

The most accepted source of clinical recommendations comes from evidence elucidated from clinical trial studies. Clinical trials are widely accepted as a unified approach to ascertaining evidence and guiding cause-and-effect studies in medical science [1]. Information from clinical trials supports and guides the selection of interventions that will be most beneficial for patients. Before information from clinical trials can be utilized, a means to assess the quality of the trial design is needed. Although clinical trials are conducted to test a hypothesis by carefully controlling for bias and confounding correlation, the quality of conclusions reached by experimental studies is dependent on statistical tests, sample sizes and significance levels. The numbers given within a clinical trial report, such as outcomes, confidence intervals, p-values, etc., are the key to identifying the strength and quality of the clinical trial [2,3]. Analysis of numerical data within the trial can improve the interpretation of statistical analysis and allow for the integration of evidence presented in different trials. However, in addition to the actual numbers, an important consideration is the surrounding context for understanding the trial and identifying potential sources of bias and error [4]. For example, reported findings may only be valid under certain conditions (e.g., specific patient population). Another obstacle that can hinder the assessment of quality is the lack of a standard form when reporting results [5,6]. The most common representation for clinical trial data and outcomes is a published clinical trial report written in narrative prose, with numbers embedded in many formats including statements, figures and tables. For a single report, numerical data is scattered throughout the paper and are often unconnected to their related context. A standard and consistent view would allow for exploring the meaning and context of data to assess the quality of a trial, improve the ability to compare and combine experiments, and bring to light assumptions and sources of error.

Ongoing research has partially examined clinical trial information. For example, the Consolidated Standards of Reporting Trials (CONSORT) statement has long researched complete and structured reporting of clinical trials [7]. The CONSORT statement consists of a 21-point checklist of required items to follow when reporting results of a clinical trial. Other groups have developed structured representations for clinical trials. The Clinical Data Interchange Standards Consortium (CDISC) establishes standards in clinical research data [8]. The Biomedical Research Integrated Domain Group (BRIDG) information model describes concepts of protocol-driven clinical research [9]. The Ontology of Clinical Research (OCRe) joins other information standards to describe human studies [10]. The Ontology for Biomedical Investigations (OBI) describes biological and medical experiments and investigations [11]. The Ontology of Scientific Experiments (EXPO) standardizes organization, execution, and analysis of a scientific experiment [12]. These efforts formalize descriptions of information within clinical trials but do not directly structure numerical data with sufficient context that can be used for the purpose of quality assessment.

In this paper, we propose a formal representation for numerical data that captures the semantic meaning and associated context of each reported number in a computable format. The goal of this representation is to establish a consistent view for answering questions related to the strength and quality of the trial. We demonstrate this structure with clinical trial reports on non-small cell lung cancer (NSCLC) with an epidermal growth factor receptor (EGFR) mutation.

## Materials and Methods

In the following sections, we describe our systematic approach to characterize different types of numerical data, delineate associated context, and present a use case and pilot evaluation.

### Locating and characterizing numerical information

**Figure 1:**
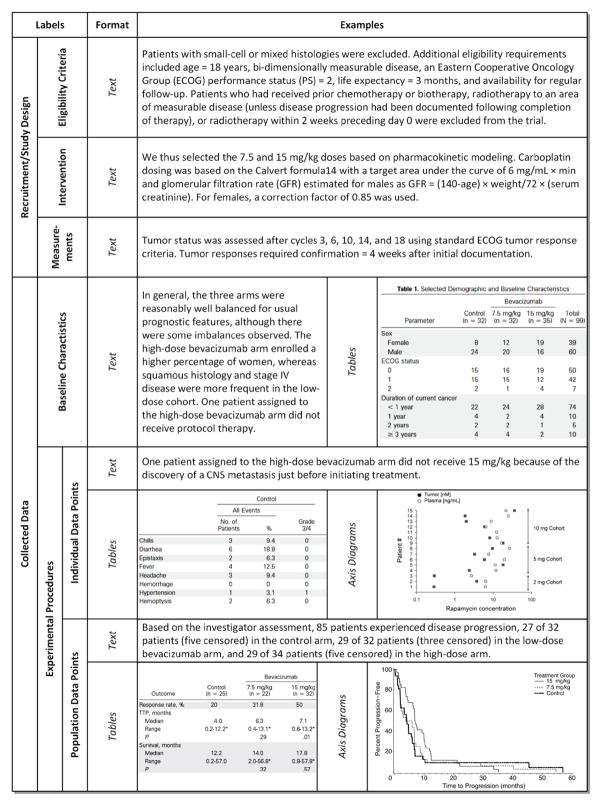
Typical examples of numerical data found in Johnson et al, organized by type. Semantic labels are shown on left (e.g., recruitment/trial design eligibility criteria). Formats are italicized (e.g., tables, text and axes diagrams). Examples of each semantic type are listed to the right of the format type.

Numbers mentioned in a clinical trial report are difficult to locate and lack standardization. We characterized the types of numbers presented in a report using a bottom-up and top-down approach. We collected ten papers on NSCLC clinical trials to learn the function of numbers within a clinical trial report. We performed a PubMed search using the keywords “phase,” “trial,” “NSCLC,” and “EGFR.” This search resulted in an initial set of ten papers. A semantic label and format type were manually assigned to each mention of a number. Semantic labels describe the numerical data’s type and where in the clinical trial report the number is presented (Figure 1, column 1). Numbers can define disease prevalence, population characteristics, estimated measurements, potential errors, and statistical analyses, such as p-values, confidence intervals, etc. For the scope of our analysis, we focused on numbers presented in the trial design/recruitment process and in the data collection process. Trial design/recruitment includes information on eligibility criteria for participants, periods of recruitment, interventions with sufficient detail, and outcomes. Collected data includes baseline information on the starting population, as well as outcomes and estimation measurements taken throughout the trial. Data are separated into baseline data about the populations and data from experimental procedures, which can be further divided into data about individual patients and data on each population. Each number was tagged with a semantic label identifying the type of number represented. In addition to the semantic labels, numerical data can take on a variety of formats, including: (i) table data; (ii) graph data, including axes, x-max, y-max, x-label, y-label, and x–y points for each series; and (iii) free-text statements (Figure 1, column 2).

### Placing numerical information into context

In our representation, the context is defined as the experimental methodology used to generate that result, the population tested, and the time-point that the estimation measurement was taken. The representation defines the context using an underlying structure based on a process model [13] and a standardized grid. The process model examines the following: Which population of patients was tested? When was this number generated? What is the meaning and significance of this number? The standardized grid is used to define and characterize the number, and addresses the following: What variable does this number measure? What are the units of this number? Together, the user can utilize the process model and standardized grid to determine the full context of the numerical data and follow the flow of the number.

#### Process Modeling

**Figure 2:**
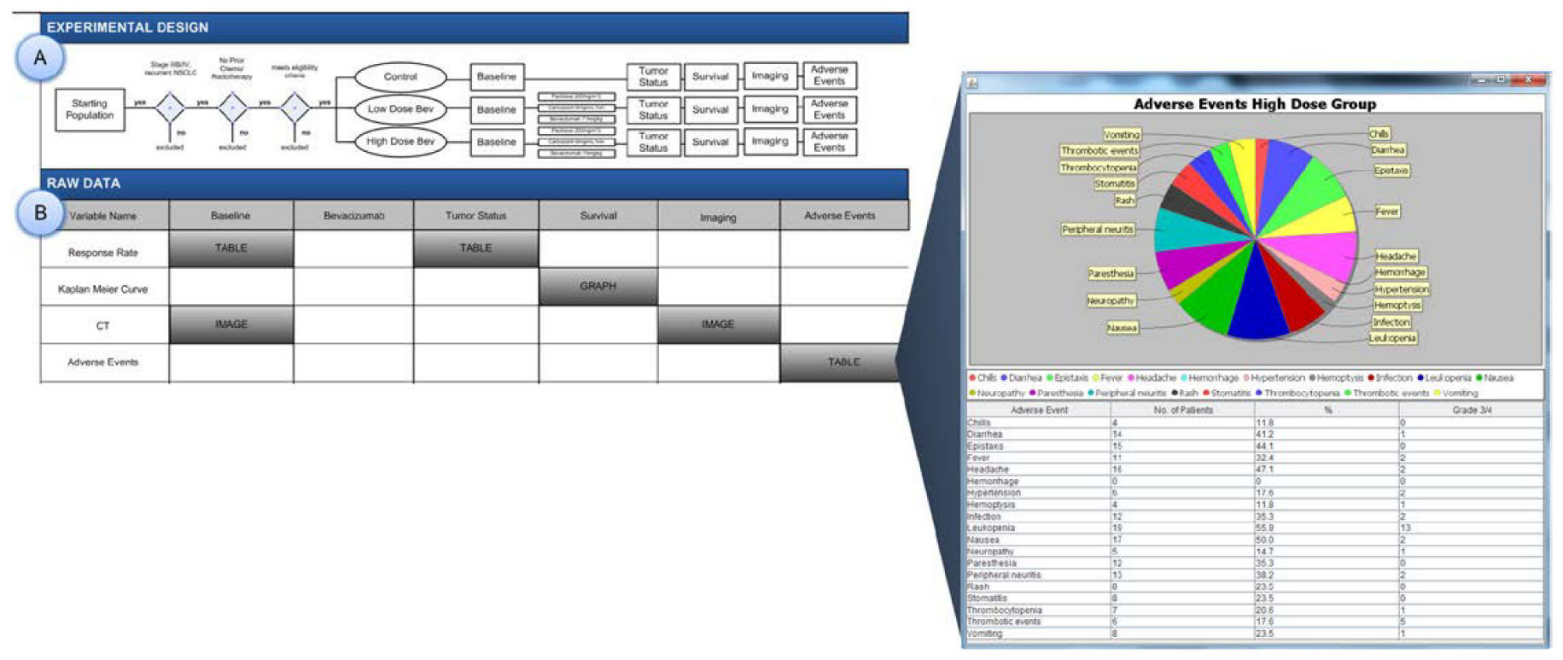
Left: (A) Visualization of the process model (B) Visualization of the standardized grid of raw data. Right: Visual display of dynamically generated pie chart of Adverse Events of the high dose group that appears when the user selects the “TABLE” button under the column labeled “Adverse Events”

The process model contains several types of building block elements (Figure 2a, left). The most common elements include: populations, eligibility criteria, and events. Ellipses are used to represent populations of individuals. Diamonds are used to represent decision nodes that affect the sample size number, such as eligibility criteria, discontinued treatment, etc. Rectangles indicate interventions and observational procedures related to hypothesis testing. Each step in the experimental procedure of a clinical trial study is labeled as an element and are linked to other elements. Example elements include genetic screenings, surgical interventions, drug cycles, imaging modalities, study end points, etc. The process model does not give a full specification of how to perform the experiment but instead gives a high level summary with enough detail to describe the origin of the number. Since elements are placed relative to other events in chronological order, the process model provides temporal relationships and can be used to generate a sequence of events. The linkages between process model element supplies back-pointers to information necessary to understand the context (e.g., population arm, the sample size of the population, randomization techniques, ascertainment methods).

#### Standardized Grid

Numerical data within the clinical trial report is organized into a spreadsheet and categorized into variables and characterizations (Figure 2b, left). We can show the relationship between numerical measurements and methodology, as the placement in the standardized grid corresponds to a variable and a process model element. Each row in the grid represents a single variable and corresponding characterization. The user can find a variable being reported, and locate its value(s) and unit(s) along the same row. The complete list of variables and its characterizations, including units, used to describe the number are drawn from existing ontologies [8–12]. For example, the variable “survival” can be associated with the following characterizations: distribution of a survival curve, the mean and median months for survival, etc. Each column in the grid represents a unique reference to an element in the process model. The content of the cell is flexible, and each cell can contain individual patient measurements, a distribution for the many characterizations of a variable, or summary statistics. Variables that are quantified at different process model steps would be displayed on the same row. For example, because tumor status includes measuring the size of the tumor at baseline before intervention and again after intervention, the variable “tumor size” and its numbers would be associated at two events points, pre-intervention and post-intervention. The grid is used to capture all the reported numbers, organizing it in a way that would facilitate secondary analysis of information. A standardized data grid allows for easier comparisons within a single trial and across different studies.

### Example Application of Visualization

A specific use case of our representation model is demonstrated for Johnson et al [14]. The process model displays the recruitment period on the left and the inventions and observations on the right (Figure 2a, left). The first node on the left is labeled as “Starting Population.” The node is connected to three diamonds, each corresponding to a separate exclusion rule. Three exclusion rules determine patients eligible for the trial. The first filter is the presence of Stage III/IV cancer, the second is no history of prior chemotherapy, and the third is a combination of other exclusion criteria. After applying these three exclusion rules, the final set of participants is obtained. In the center, this set of participants is randomized into three study arms. Each row in the process model represents its own study arm. In our running example, the study arms are control, low dose, and high dose. Following randomization, the right side displays experimental procedures referred to as a sequence of events. The sequence of events branch from each population node displaying only the interventions specific to each study arm. For our running example, interventions were either 7.5 mg/kg Bevacizumab, 15 mg/kg Bevacizumab or no drug. After intervention, tumor status was measured, following that was survival, and lastly, adverse events.

Clicking on “Adverse Events” under the high dose population in the data grid (Figure 2b, left) displays the adverse events for the high dose study arm (Figure 2, right). A pie chart is generated along with a display of the raw numbers.

The advantage of storing numbers in their raw form is the ability to randomly generate charts and graphs. Thus by using our representation, we are able to visually display the meaning of the number within the context of the clinical trial report.

### Evaluation of System

We compared two situations, normal status quo analysis of clinical trial reports and analysis with the newly developed structured representation, to test the claim that the representation is beneficial. The evaluation consists of a guided demonstration and standard questions for radiologists, clinicians, and bio-statisticians during journal club meetings. Four clinical trial reports were given to six researchers – 2 graduate students, 2 biostatisticians, and 2 principal investigators of clinical trials. Each researcher received two reports in the status quo form and two reports as structured representations. For the status quo, researchers were allowed to manually interpret and synthesize the information as they saw fit. To account for varying levels of difficulties in clinical trial reports, we randomized the studies given in standard form versus representation form for each researcher and standardized results with an internal control. In other words, we reported the results with representation relative to the results without representation for each clinical trial separately, and then averaged this value over all clinical trials. During the evaluation, researchers were asked to answer 10 yes/no questions in each of the three areas: a) characteristics of the trial, b) quality of the trial, and c) significance of the science. Questions were developed by a senior biostatistician to assess major issues related to understanding the quality of a clinical trial. A biostatistics professor reviewed the questions to ensure that they conformed to items in CONSORT. The evaluation compared time taken to complete each section and accuracy of the answers. We followed up with an open-ended interview to discuss other factors contributing to the strengths and shortcomings of the visualization.

## Results

### Evaluation of Visualization

We found that researchers were able to answer questions faster and with better accuracy when given the representation. When comparing accuracy within each researcher, we found that having the proposed hybrid representation when answering questions yielded a 12.7% higher accuracy and was on average 25.9% faster (excluding one extreme outlier) than not having the representation. Questions pertaining to characteristics of the trial, such as locating numerical data, were quickly and accurately answered when using the representation. We noticed that one paper had similar accuracy when comparing between having and not having the representation. Though accuracy was similar, the time used to obtain these answers were up to twice as long when not having the representation. These results favor the representation as a tool to save time. Further evaluation is needed to determine added features to allow for a more helpful and informative visualization.

In interviews, users all agreed that the tool was helpful. They said the representation can aid with comparisons of study arms in different trials, and one researcher would like that functionality added. Researchers appreciated the appropriate level of detail and the intuitiveness of the process model. The process model of this representation differs from other workflow models in that it abstracts only enough information to interpret numerical data and does not contain steps for replicating the trial. Researchers involved in the clinical setting expressed a willingness to use this tool to extrapolate patient outcomes for similar patients. All researchers were in favor of further development to extend functionality, such as having a toolbox to perform statistical operations and integration of data from multiple trials. Initial studies showed promising results. Currently, we are undertaking a formal evaluation with a larger sample size to test our hypothesis that having the representation yields a significant difference in time and accuracy.

## Discussion

We describe our efforts to design a representation, a novel hybrid between a process model and spreadsheet grid, to create a consistent and comprehensive display of numbers presented in clinical trial reports. We propose a tool to visualize numbers within a clinical trial report and explore its context. The representation is generalizable as the process model can be built to accommodate any level of detail, and the data grid is adaptable and based off of existing ontologies. By portraying the interventions in the trial as a sequence of events, the process model is then mapped in sequential order, and users can visually validate the trial design and describe temporal relationships. A standard representation would lead to a familiarity with identifying important details related to understanding statistical significance and scientific discoveries. This understanding would allow computerized clinical decision support systems to assess the internal validity of the study, reason on the evidence, and generate recommendations tailored to a patient’s individual needs and characteristics.

One limitation is the ability to view only one clinical trial report at a time. An effort to develop an integrated visualization for multiple clinical trial reports is underway. With the integration of data, issues arise pertaining to appropriate ways for dealing with conflicting data and assigning relative weights to data. Another limitation is the inability to perform statistical tests with the abstracted numbers. Because our abstractions come from clinical trial reports, the inability to access the raw data from the clinical trial hinders our ability to freely perform statistical tests on the fly. Another limitation is the use of a pilot evaluation to demonstrate the usability of the representation and visualization. While initial results are promising, a larger more comprehensive evaluation is underway.

Future work includes continuing development of the representation, generating new views of the data to summarize studies, and producing a global visualization of a disease using clinical trial results. We are developing informatics tools to capture numerical data from published clinical trial reports using natural language processing (NLP) methods [15]. This system involves a backend data model to create the visualization. The proposed implementation will consist of a graphical abstraction tool that guides a user through populating the data model with relevant findings drawn from the clinical trial report and an end-user visualization that displays information from the data model.

## Conclusions

Currently, there is no means of modeling the quantitative information in clinical trial reports. Several efforts have gone into structuring information for the purpose of information retrieval but not for assessing the quality of clinical trial results. We demonstrate a formal representation and visualization to describe numerical data within a clinical trial report. Our representation organizes and compartmentalizes this information by capturing the context and meaning of each number. These efforts are important to assess the quality of results and integrate evidence from multiple clinical trials studies.

## References

[1] Horwitz RI (1987). The experimental paradigm and observational studies of cause-effect relationships in clinical medicine. J Chronic Dis.

[2] Pan G, Ke S, Zhao J (2013). Comparison of the efficacy and safety of single-agent erlotinib and doublet molecular targeted agents based on erlotinib in advanced non-small cell lung cancer (NSCLC): a systematic review and meta-analysis. Target Oncol.

[3] Chootrakool H, Shi JQ, Yue R (2011). Meta-analysis and sensitivity analysis for multi-arm trials with selection bias. Stat Med.

[4] Mills EJ, Ioannidis JP, Thorlund K, Schünemann HJ, Puhan MA, Guyatt GH (2012). How to use an article reporting a multiple treatment comparison meta-analysis. JAMA.

[5] Jüni P, Altman DG, Egger M (2001). Systematic reviews in health care: Assessing the quality of controlled clinical trials. BMJ.

[6] Chalmers TC, Smith H, Blackburn B, Silverman B, Schroeder B, Reitman D, Ambroz A (1981). A method for assessing the quality of a randomized control trial. Control Clin Trials.

[7] Moher D, Jones A, Lepage L, CONSORT Group (Consolitdated Standards for Reporting of Trials) (2001). Use of the CONSORT statement and quality of reports of randomized trials: a comparative before-and-after evaluation. JAMA.

[8] The Clinical Data Interchange Standards Consortium (CDISC) (2013). http://www.cdisc.org.

[9] The Biomedical Research Integrated Domain Group (BRIDG) (2013). http://www.bridgmodel.org.

[10] Sim I, Carini S, Tu S, Wynden R, Pollock BH, Mollah SA, Gabriel D, Hagler HK, Scheuermann RH, Lehmann HP, Wittkowski KM, Nahm M, Bakken S (2010). The human studies database project: federating human studies design data using the ontology of clinical research. AMIA Summits Transl Sci Proc.

[11] Brinkman RR, Courtot M, Derom D, Fostel JM, He Y, Lord P, Malone J, Parkinson H, Peters B, Rocca-Serra P, Ruttenberg A, Sansone SA, Soldatova LN, Stoeckert CJ, Turner JA, Zheng J, OBI consortium (2010). Modeling biomedical experimental processes with OBI. J Biomed Semantics.

[12] Soldatova LN, King RD (2006). An ontology of scientific experiments. J R Soc Interface.

[13] De Carvalho ECA, Jayanti MK, Batilana AP, Kozan ZMO, Rodrigues MJ, Shah J, Loures MR, Pietrobon R (2010). Standardizing clinical trials workflow representation in UML for international site comparison. PLoS ONE.

[14] Johnson (2004). Randomized phase II trial comparing bevacizumab plus carboplatin and paclitaxel with carboplatin and paclitaxel alone in previously untreated locally advanced or metastatic non-small-cell lung cancer. J Clin Oncol.

[15] Hsu W, Speier W, Taira RK (2012). Automated extraction of reported statistical analyses: Towards a logical representation of clinical trial literature. Proc AMIA Fall Symp.

